# *Centaurea behen* leaf extract mediated green synthesized silver nanoparticles as antibacterial and removing agent of environmental pollutants with blood compatible and hemostatic effects

**DOI:** 10.1038/s41598-024-64468-9

**Published:** 2024-06-17

**Authors:** Mohadese Abdoli, Salar Khaledian, Maryamosadat Mavaei, Pouria Hajmomeni, Mahnaz Ghowsi, Farshad Qalekhani, Houshang Nemati, Ali Fattahi, Komail Sadrjavadi

**Affiliations:** 1 Nano Drug Delivery Research Center, Health Technology Institute, Kermanshah University of Medical Sciences, Kermanshah, Iran; 2 Department of Nanobiotechnology, Faculty of Innovative Science and Technology, Razi University, Kermanshah, Iran; 3 Infectious Diseases Research Center, Health Institute, Kermanshah University of Medical Sciences, Kermanshah, Iran; 4 Student’s Research Committee, School of Pharmacy, Kermanshah University of Medical Sciences, Kermanshah, Iran; 5 Pharmaceutical Sciences Research Center, Health Institute, Kermanshah University of Medical Sciences, Kermanshah, Iran; 6 Department of Biology, Faculty of Science, Razi University, Kermanshah, Iran; 7 Medical Biology Research Center, Health Technology Institute, Kermanshah University of Medical Sciences, Kermanshah, Iran

**Keywords:** Antibacterial activity, Catalytic activity, Hemolytic activity, Silver nanoparticles, Green synthesis, Biotechnology, Nanoscience and technology

## Abstract

The present study focused on evaluating the antibacterial properties, radical scavenging, and photocatalytic activities of *Centaurea behen-mediated* silver nanoparticles (Cb-AgNPs). The formation of Cb-AgNPs was approved by UV–Vis spectrometry, Fourier-transform infrared spectroscopy, transmission electron microscopy, scanning electron microscopy (SEM), energy dispersive X-ray and X-ray diffraction. The results showed that the obtained AgNPs have a maximum absorbance peak at 450 nm with spherical morphology and an average size of 13.03 ± 5.8 nm. The catalytic activity of the Cb-AgNPs was investigated using Safranin O (SO) solution as a cationic dye model. The Cb-AgNPs performed well in the removal of SO. The coupled physical adsorption/photocatalysis reaction calculated about 68% and 98% degradation of SO dye under solar irradiation. The Cb-AgNPs inhibited the growth of gram-negative or positive bacteria strains and had excellent DPPH radicals scavenging ability (100% in a concentration of 200 µg/ml) as well as a good effect on reducing coagulation time (at concentrations of 200 and 500 µg/mL reduced clotting time up to 3 min). Considering the fact that green synthesized Cb-AgNPs have antioxidant and antibacterial properties and have a good ability to reduce coagulation time, they can be used in wound dressings. As well as these NPs with good photocatalytic activity can be a suitable option for degrading organic pollutants.

## Introduction

Silver compounds have been utilized in medicine for a long time to prevent and treat bacterial infections. This is due to silver's superior antifungal, antibacterial, and antiviral properties compared to other metals like gold, titanium, zinc, and copper^1,2^. The silver ions released from the silver-containing substrates react with the thiol groups of enzymes and proteins that are important for the bacterial cells, and through disruption in their functions, exterminate bacteria^3,4^. Silver nanoparticles (AgNPs) which release silver ions gradually have recently been developed to be a promising antibacterial agent^5,6^. AgNPs exhibit a high surface-to-volume ratio, making them more efficient than bulk silver metal due to the large number of surface atoms^7^. However, producing pure AgNPs is costly and poses environmental risks. Currently, the biosynthesis of AgNPs through green chemistry is gaining attention among researchers. This method involves using plant extracts, which offer the benefit of being naturally biodegradable^8,9^. The biological reduction of metals using plant extracts has been recognized since the early 1900s^10,11^.

In spite of the antimicrobial, anti-inflammation, anti-biofilm, and anti-viral properties and enhanced wound healing properties of AgNPs, their small size allows them to translocate into the bloodstream during inhalation, oral administration, or injection. This can lead to interactions with red blood cells (RBCs) and other blood components, potentially triggering pathophysiological conditions. Therefore, considerate the interaction of AgNPs with RBCs and coagulation is vital^12^. Primary homeostasis and blood clotting play axial roles in mediating body immunity. Although previous blood studies have examined the compatibility of AgNPs, all materials used to make AgNPs vary in size, coating, and concentration^13–15^.

Previous studies have shown that coated AgNPs cause hemolysis in a dose-dependent manner by changing the membrane integrity and surface characteristics. It has also been shown that the size of AgNPs has a significant effect on their hemolytic properties and therefore a suitable size and dosage of these nanoparticles can moderate hemolytic response^16,17^.

Like gold nanoparticles, in addition to being involved in wound healing and biosensor development, AgNPs are also used as catalyst for detoxification of various pollutants^18–20^. It is reported that green synthesized AgNPs have catalytic activity for degrading of dyes as organic pollutants^21^. As various industries continue to expand, environmental pollution is also increasing proportionally^22,23^. In particular, the entry of many pollutants into the water causes irreparable environmental hazards^24^. Many researchers have reported the use of available, inexpensive, and natural adsorbents (like algae, bagasse, etc.) to remove contaminants and heavy metals^25–27^. Various methods have been introduced to remove contamination from water, of which catalytic reaction is one of the most important and cost-effective methods^28^. With the advancement of nanoscience, various metal nanoparticles, including gold and silver, are being utilized as nano catalysts^29^. Recently, the catalytic activity of AgNPs for the degradation of specific aqueous dyes has been reported^30,31^.

So, in the present study we synthesized AgNPs using aqueous plant extract of *Centaurea behen* as a reducing and stabilizing agent. *C. behen* is an annual or perennial herb that has been traditionally used to treatment of jaundice, cystic fibrosis, kidney stones, etc^32^. This medicinal plant contains numerous flavonoids and terpenoids, and its antioxidant and cytotoxic properties have been evaluated in previous studies^33,34^. Therefore, in this work, we synthesized and characterized C. behen mediated AgNPs (Cb-AgNPs) using UV–visible spectrophotometer, Fourier-transform infrared spectroscopy (FTIR), scanning electron microscopy (SEM) with energy dispersive X-Ray (EDX) detector X-ray diffraction (XRD) and transmission electron microscopy (TEM).

However, until now, *C. behen* has not been used for AgNPs synthesis, and to the best of our knowledge, this is the first time in the present study that *C. behen* leaf extract has been used for green synthesis of AgNPs. The overall aim of this article is to examine photocatalytic performance, antimicrobial and antioxidant properties of Cb-AgNPs. Furthermore, effects of Cb-AgNPs on clotting time and hemolysis were evaluated.

## Results and discussion

### Physicochemical analyses

**Figure 1:**
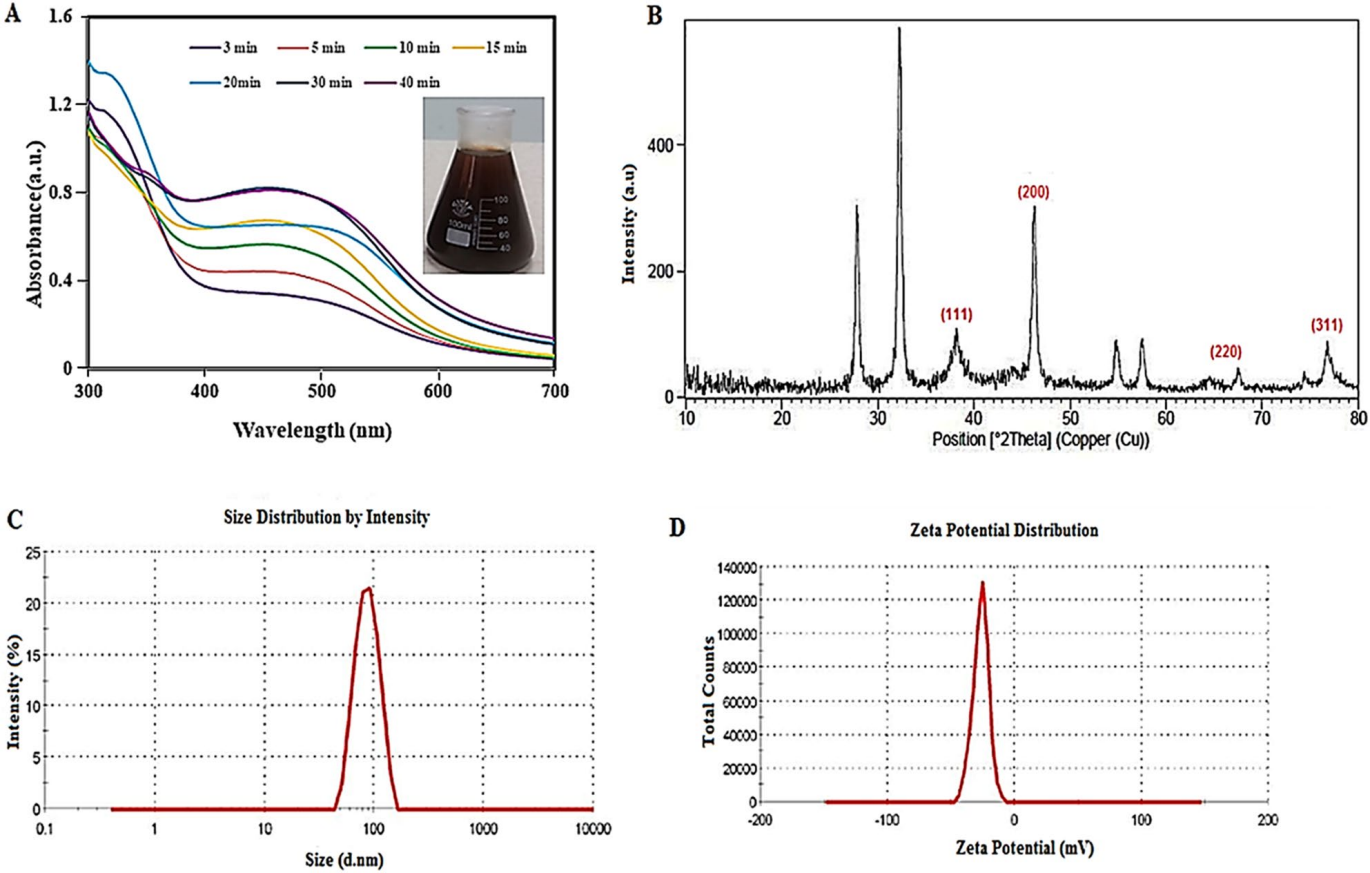
(**A**) UV–Vis absorption spectrum, (**B**) XRD pattern, (**C**) DLS profile, and (**D**) zeta potential of synthesized Cb-AgNPs using *C. behen* extract.

The moisture content (MC) of *C. behen* leaves was obtained 22.5 ± 0.46%. After several minutes’ exposure to sunlight, the aqueous AgNO_3_ solution became dark brown. UV–vis spectroscopy as a fast and low-cost technique has been used for initial approval of Cb-AgNPs’ synthesis and determination of optimum reaction time; the presence of a band at 450 nm confirmed the synthesis, and the reduction was almost completed within 30 min. The UV–Vis spectra of the samples are shown in Fig. 1A.

The appearance of brown color and absorbance band at 450 nm verify the reduction of Ag^+^ to Ag^0^ and the formation of AgNPs by Cb-extract. In fact, plant metabolites containing hydroxyl groups can act as reducing agents (Ag^+^ $\to$ Ag (0)) in the presence of sunlight^35^. Previous study indicated flavonoids enrichment of Centaurea species^36^. Flavonoids and polyphenols, as plant secondary metabolites with the poly hydroxyl group, play roles in the reduction of metal ions into nanoparticles^37^. This reaction related to their electron or hydrogen atoms donation capability; the functional hydroxyl groups of flavonoids are significantly involved in scavenging the free radicals or chelating the metal ions^38–40^. For this reason, the formation of AgNPs using C. behen extract is likely due to the electron transfer from the oxidized form to the reduced form of silver ions^41^.

Figure 1B shows the XRD patterns of the AgNPs synthesized by *C. behen*. The 2θ peaks observed at 38.2°, 46.2°, 64.6°, and 76.7° corresponds to (111), (220), (200) and (311) planes of Bragg's reflection of silver that confirmed the formation of face-centered cubic (FCC) silver crystal, which is also in accordance with the earlier studies^42,43^. Also, a few unmarked peaks in the XRD pattern of Cb-AgNPs appeared at 27.8, 32.2, 54.8, and 57.4, which may be associate with the presence of bio-organic ingredients of the plant extract in the shell of NPs^44,45^.

The hydrodynamic diameter of the biosynthesized Cb-AgNPs determined by DLS (Fig. 1C), whereas TEM represents the actual diameter of the nanoparticles because it measures the sample at the dry state. The average hydrodynamic size (diameter) of the Cb-AgNPs is 96.67 nm polydispersity index (PDI) (PDI = 0.235), and their zeta potential value was − 26.6 mV (Fig. 1D). The high negative value of nanoparticle confirms the stability of synthesized AgNPs^46^.

**Figure 2:**
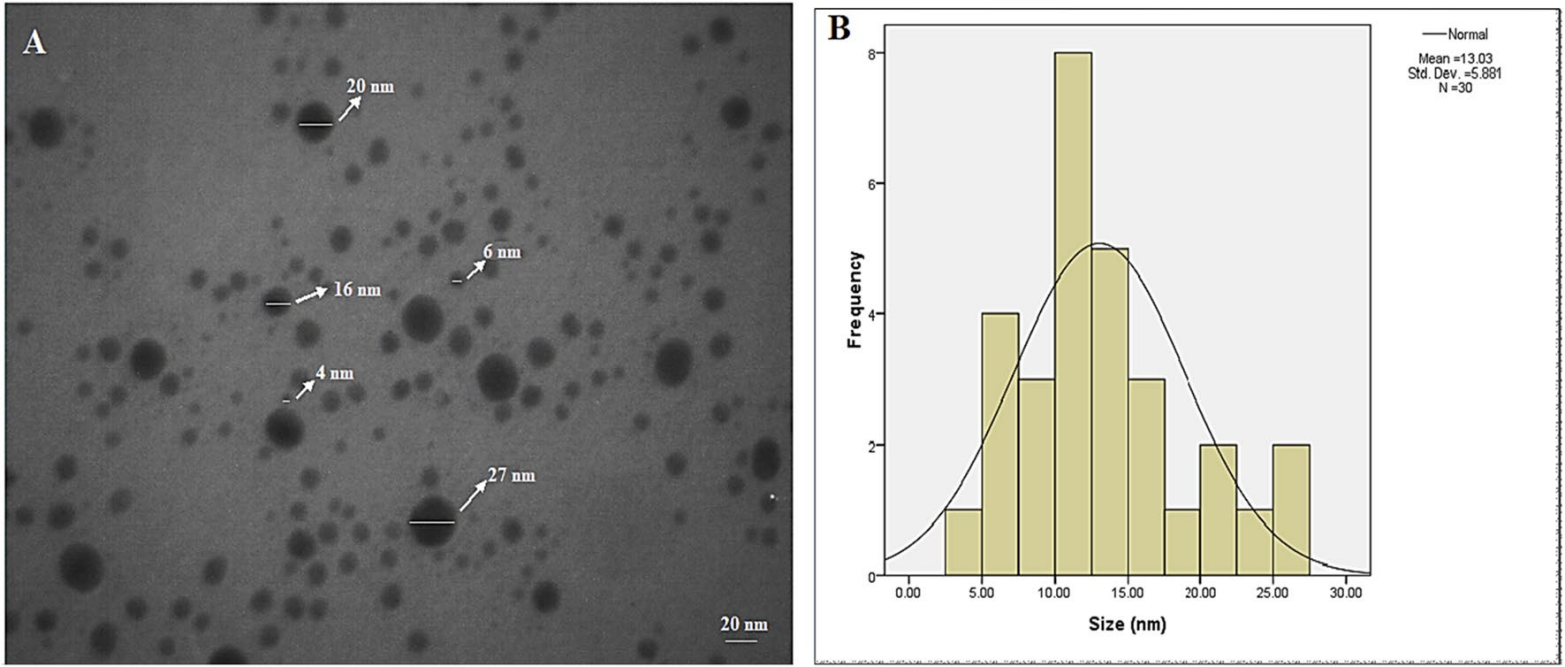
(**A**) TEM images associated with the AgNP’s synthesized by *C. behen* extract and (**B**) particle size distribution. AgNPs with spherical morphologies and well-dispersed can be appreciated.

The shape, size, and morphology of the AgNPs were analyzed using TEM (Fig. 2A and B). The TEM image showed that the particles are in a range of 8–24 nm and with the average size of 13.03 nm and clearly illustrated that the particles are spherical.

**Figure 3:**
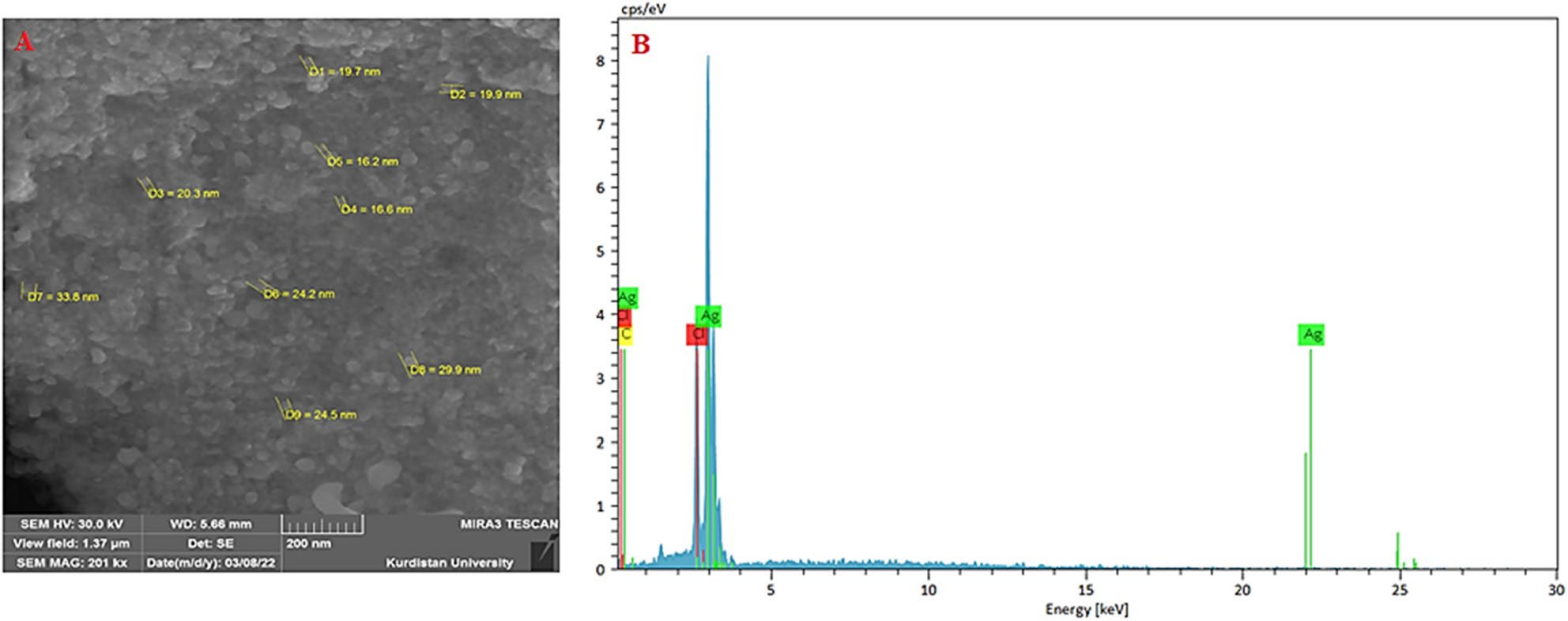
The SEM micrograph (**A**), and EDX pattern (**B**) of synthesized Cb-AgNPs.

The SEM analysis was utilized for visualizing the surface morphology and particles’ sizes of Cb-AgNPs. Figure 3A showed the SEM image of the synthesized Cb-AgNPs. The size of the capped Ag nanoparticles was in the range of between 16.2 and 33.8 nm and the shapes were spherical and some are irregular, which confirm the results of TEM and DLS. Based on the results shown in Fig. 3, the size control and morphology of Cb-AgNPs may be affected by the interactions between reducing biomolecules and metal atoms^47,48^, which in this study is *Centaurea behen* as sesquiterpene lactones. Figure 3B, showed Three elements silver, carbon and chlorine have been detected in the EDS spectrum taken. The presence of carbon indicates that the surfaces of silver nanoparticles are covered by the metabolites in the *Centaurea Behen* Leaf extract.

**Figure 4:**
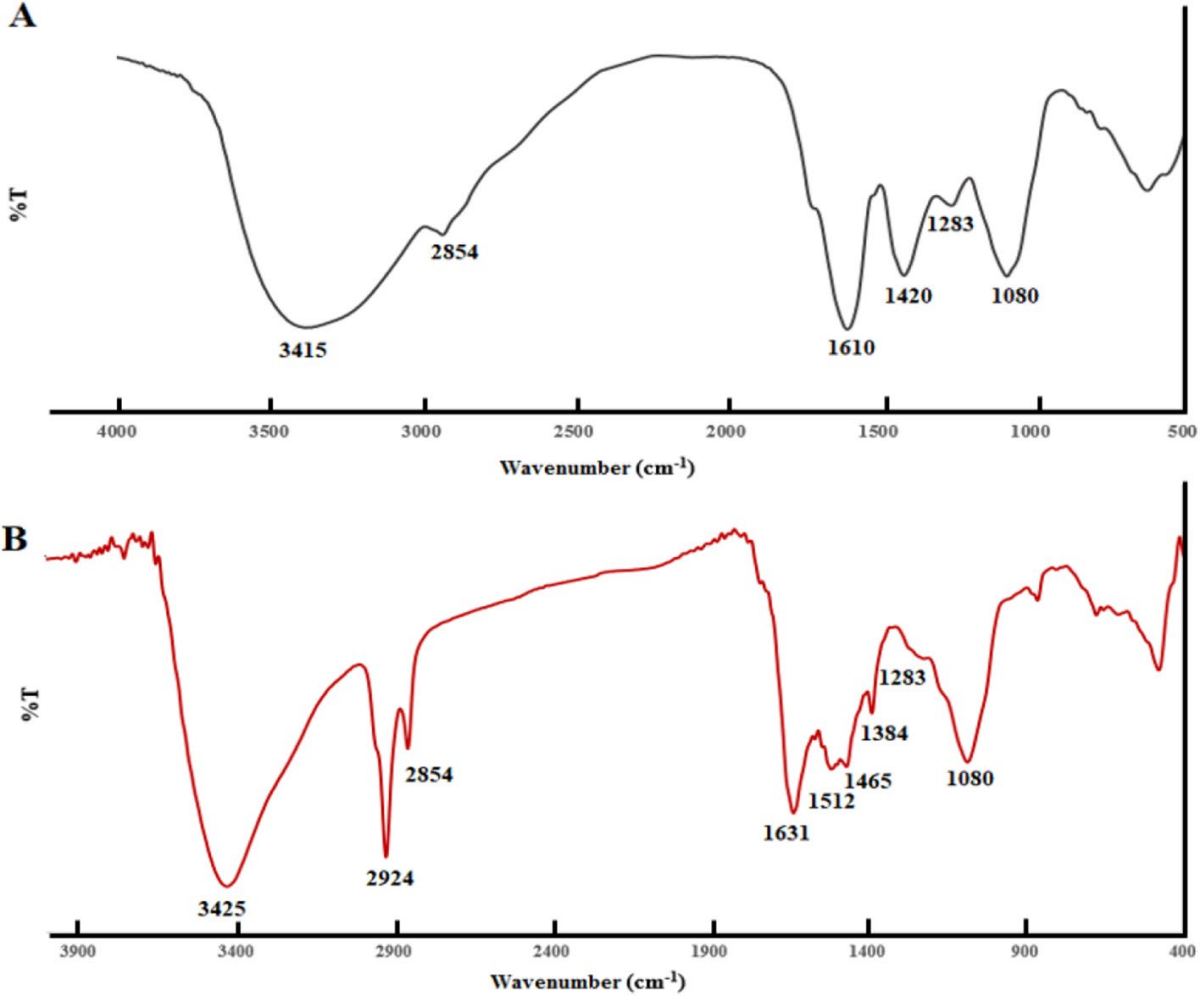
(**A**) FTIR spectrum of *C. behen* extract and (**B**) synthesized AgNPs.

The FTIR spectrum of plant extract and synthesized AgNPs was showed in Fig. 4. The main band of plant extract including 3415, 1610, 1420, 1283 and 1080 cm^-1^ (Fig. 4A). The characteristic band of obtained AgNPs (Fig. 4B) was as follows: 3425 (O–H stretching of alcohols or phenols), 2924 and 2854 (O–H stretching and C–H stretching), 1631 (C=C stretching and N–H bending of amines), 1512 (N–O asymmetric stretching), 1465 (C–C stretching), 1384 and 1080 cm^-^1 (C–O stretching), 1283 (C–N stretching). The band at 1631 and 1512 cm^−1^, propose the presence of proteins on the surface of Cb-AgNPs. Furthermore, the bands related to the C=C and C–O indicated that in addition to protein, other compounds, such as alkaloids and flavonoids, are present on the surface of Cb-AgNPs.

### Effect of operating parameters on the Adsorption/photo-catalytic degradation of Safranin O dye

Unfortunately, the significant rise in water pollution caused by various contaminants including inorganic pollutants, new human-induced substances, and emerging organic contaminants has become a serious challenge^49–51^.

One of the most effective methods for removing hazardous, toxic organic pollutants from the environment, particularly from wastewater, is photocatalytic degradation using catalyst particles. Among various nano photocatalyst materials, AgNPs have received significant attention due to their high photocatalytic activity, low cost, non-toxicity, and high stability in aqueous solutions^52–55^.

To understand photocatalytic degradation of Safranin O (SO) dye using Cb-AgNPs as catalyst, we studied the influence of several factors on the adsorption/photo-catalytic process including contact time, initial concentration of SO dye, amount of photocatalyst, solution pH and temperature.

**Figure 5:**
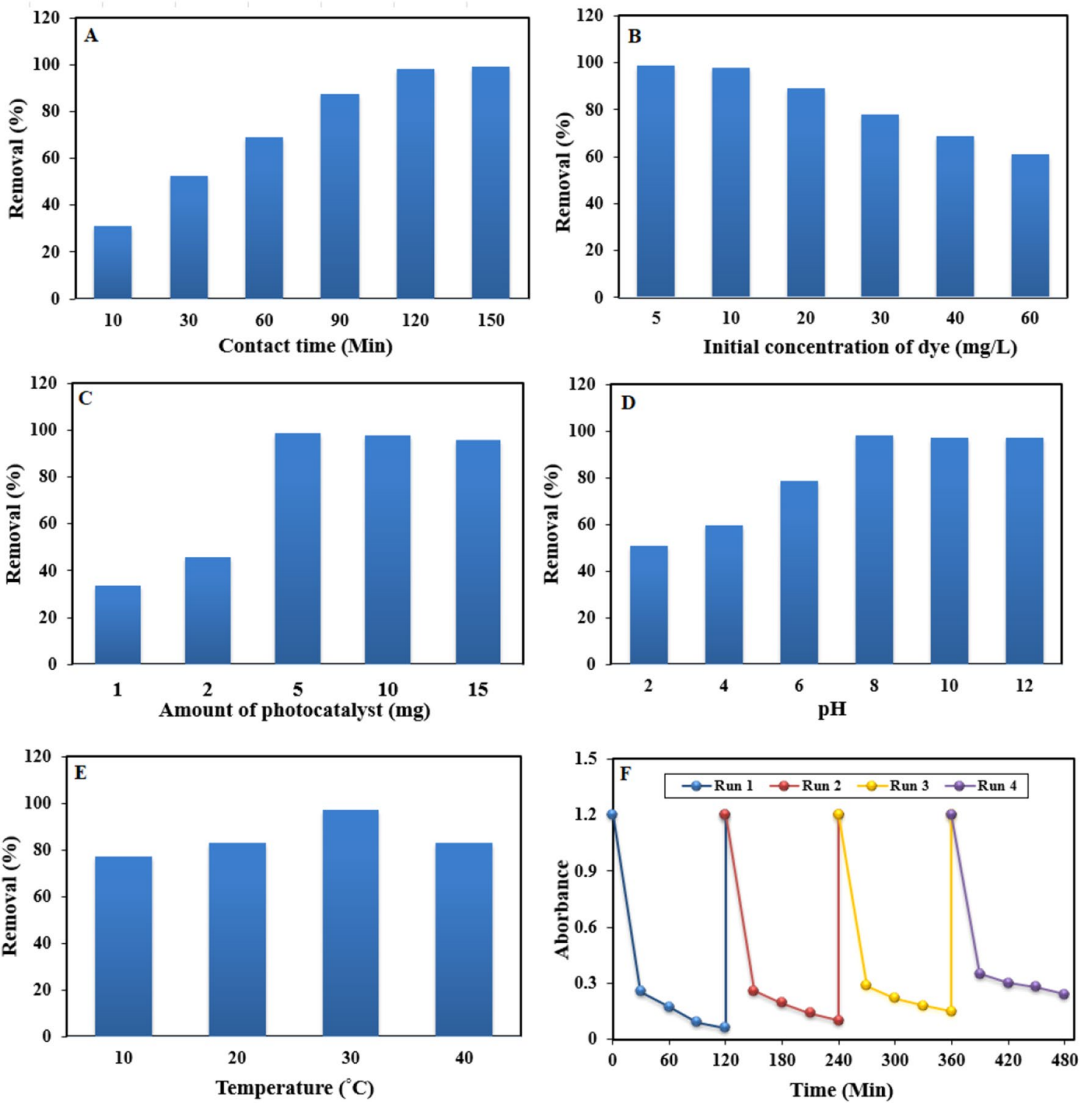
Effects of considered factors on Safranin removal efficiency, (**A**) Effect of contact time, (**B**) effect of initial SO dye concentration, (**C**) effect of photocatalyst dosage, (**D**) effect of pH, (**E**) effect of temperature and (**F**) reusability of the Cb-AgNPs after four successive runs.

Adsorption of SO dye on Cb-AgNPs surface, obviously, is a function of the contact time, which is corresponding to the time of adsorption, or the state of saturation of the absorbent surface by the absorbate (SO dye). Figure 5A presents the effect of contact time on the removal of SO using 5 mg of catalysts in aqueous solution. Six samples were used to oversee the effect of contact time at 10, 30, 60, 90, 120, and 150 min. The optimum contact time was 120 min for Cb-AgNPs.

The effect of initial SO dye concentration on its degradation has been studied with varying concentration of SO dye from 5 to 60 mg/L, keeping the Cb-AgNPs concentration set at 5 mg (Fig. 5B). It is found that the degradation efficiency of Cb-AgNPs sample has been increased initially for dye concentration up to 10 mg/L for SO and then decreased**.** Hence, the efficacy of dye removal could be enhanced by reducing the initial concentration of the dye. This can be attributed to the increased adsorption of dye molecules on the photocatalyst surface with higher initial dye concentrations. As more active sites become occupied by dye molecules, the adsorption of O_2_ and OH on the photocatalyst decreases, resulting in reduced regeneration of radicals. Additionally, higher dye concentrations lead to the blocking of photons before reaching the photocatalyst surface, thereby decreasing photon adsorption by the photocatalyst^56^.

Optimal catalyst dosage plays a crucial role in enhancing the rate of electron/hole pair generation and consequently the production of OH⋅ radicals, which are essential for achieving effective photodegradation efficiency. Therefore, the impact of catalyst dosage on the degradation efficiency was investigated within the range of 1 to 15 mg while maintaining the initial dye concentration at 10 mg/L (Fig. 5C). As depicted, a catalyst amount of 5 mg exhibited the highest efficiency in decolorizing SO, followed by a sharp decline in decolorization efficiency. The increase in catalyst dosage resulted in the creation of more active sites on the photocatalyst surface, leading to enhanced hydroxyl radical formation. However, an adverse effect was observed with further increases in catalyst dosage, attributed to particle aggregation causing a screening effect that hinders photon access to the inner catalyst surface. Moreover, excessive catalyst dosage reduces light penetration due to the protective effect of suspended particles, thereby diminishing the photodegradation rate^56^.

The adsorption of SO dye on Cb-AgNPs was examined across the pH range of 2–12. It was observed that the adsorption percentage increased as the pH rose from 2 to 8, reaching a plateau thereafter (Fig. 5D). This trend can be explained by the abundance of H^+^ ions in strongly acidic conditions (pH 2), which compete with the positively charged dye molecules for active sites on the Cb-AgNPs surface, resulting in reduced adsorption. As the pH increased towards 8, the depletion of H^+^ ions led to enhanced interaction between the surface and the dye molecules. Beyond pH 8, the surface acquired negative charges, facilitating stronger interactions with the positively charged dye molecules, thus increasing adsorption. This confirms that the electrostatic attraction is the responsible force for dye adsorption^57^.

The temperature is recognized as one of the influential factors in the adsorption process. To investigate its effect on the removal efficiency of SO, experiments were conducted at a constant adsorbent dose of 5 mg and an initial concentration of 10 mg/L (Fig. 5E). The results indicate that the adsorption percentage of the SO dye increases with rising temperature from 10 to 30 °C. However, beyond this temperature, the removal percentage begins to decline, suggesting that the adsorption of SO onto the surface of the adsorbent particles is favored at lower temperatures and is governed by an exothermic process. This could be attributed to the weakening of attractive forces between the dye molecules and the surface of the particles^58^.

In order to examine the reusability and durability of as prepared Cb-AgNPs photocatalyst, a recycling study of Cb-AgNPs was carried out with a stock solution of SO under identical conditions. Hence, the stability of the Cb-AgNPs was tested for successive four recycling runs. As shown in Fig. 5F, the reusability of the Cb-AgNPs photocatalyst was demonstrated up to a fourth cycle run with a low decrease in photo-degradation efficiency, i.e., 98% to about 83% in the first to fourth cycle runs. The results presented in Fig. 5F demonstrate that the Cb-AgNPs possess robust and excellent cycle stability.

The removal efficiency of Cb-AgNPs was compared with those of other photocatalysts reported in literature, and the results are listed in Table 1. From the results, it can be seen that the degradation efficiency of Cb-AgNPs is same or higher than that of other photocatalysts. Hence, Cb-AgNPs could be acted as an excellent photocatalyst in the application for the removal of organic pollutants.

**Table 1:** Comparison with other photocatalysts for the removal of SO dye.

Sample	Dye concentration	Contact time	pH	Dosage of catalyst	Removal efficiency%	References
CMC-g-poly (AA-co-IA)	10 mg/L	40 min	8	2 g/L	99.78	^59^
PUCP1@rGO coordination polymer	5 μM	90 min	7	10 mg	76	^60^
TiO_2_/IO_4_^−^	10 mg/L	50 min	6	0.4 g /L	99	^61^
Quaternary LaNiSbWO4-G-PANI polymer nanocomposite	1 × 10^–4^ M	120 min	–	0.1 g	84	^62^
Holmium-doped titanium dioxide (Ho-TiO_2_)	20 mg/L	180 min	8	0.035 g	87	^63^
Titania coated silica nanocomposite	17.61 mg/L	12 min	6.2	89.80 mg/g	93.29	^64^
ZnS NPs	1.0 × 10^−5^ M	40 min	7	–	51	^65^
Cb-AgNPs	10 mg/L	120 min	8	5 mg	98	This work

### Adsorption/photo-catalytic performance of Cb-AgNPs

**Figure 6:**
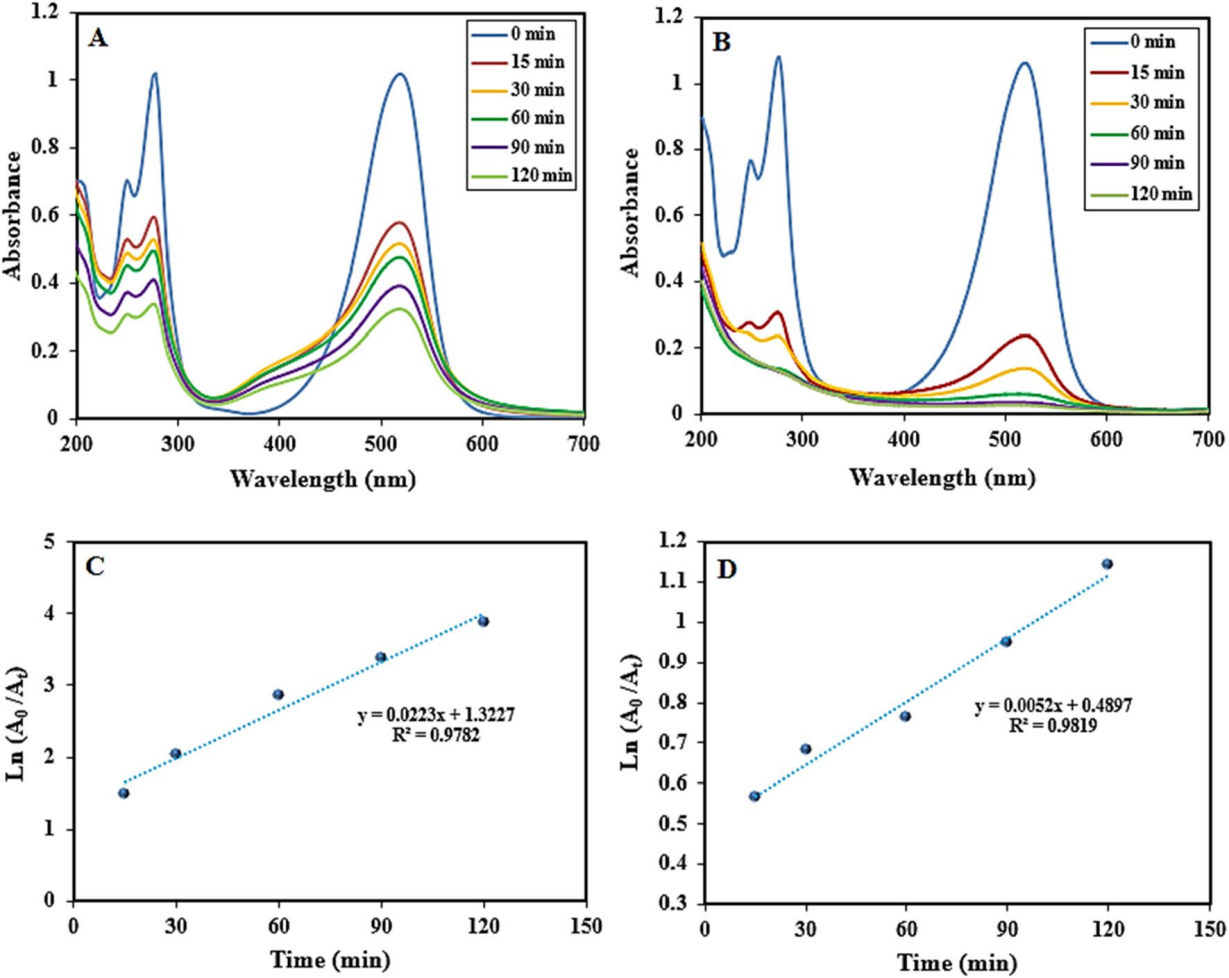
Spectra of SO dye: (**A**) physical adsorption in dark condition followed by photocatalysis procedure and (**B**) coupled physical adsorption/photocatalysis procedure; pseudo-first-order kinetics plot for photo-degradation of SO dye: (**C**) physical adsorption in dark condition followed by photocatalysis procedure and (**D**) coupled physical adsorption/photocatalysis procedure in the presence of Cb-AgNPs.

This section focuses on the synergistic influence of physical adsorption of SO dye on Cb-AgNPs and their application toward the photocatalytic degradation of SO under sunlight irradiation^66^. For further identification of the photocatalysis performance of Cb-AgNPs, other processes including adsorption in dark conditions followed by photocatalysis and coupled physical adsorption/photocatalysis of SO dye under sunlight illumination were evaluated, and the results are presented in the Fig. 6. Before sunlight irradiation, the SO dye solution containing Cb-AgNPs was kept in the dark conditions and stirred for 60 min to establish adsorption/desorption equilibrium. Then the mixture was placed under sunlight irradiation 120 min more for further photo-degradation procedure and to absorb free dye molecules presented in the solution. According to the obtained results, only 42% SO dye molecules were adsorbed by Cb-AgNPs under dark conditions and 68.33% of SO dye molecules degraded in sunlight Fig. 6A. Maybe the surface of Cb-AgNPs as a photocatalyst was covered and saturated by SO dye molecules, and photodegradation process was suppressed, but electron/hole pairs (e^−^/h^+^) were generated when solution was irradiated under sunlight, which reacted with water to generate and release hydroxyl and superoxide radical anions on the photo-catalyst surface^67^. As a result, the superoxide radicals break the conjugation of the dye molecules. In the coupled procedure, we supposed simultaneous adsorption of SO dyes onto the Cb-AgNPs surface and generation of e^−^/h^+^ pairs on the absorption of sunlight. Then free radicals generated disrupt conjugation of the adsorbed dye and free dye molecules in the solution and finally degraded the SO dye molecules^68,69^. The photocatalytic degradation percentage of SO by Cb-AgNPs was found to be 98% in 120 min of photo-irradiation, as is displayed in Fig. 6B. The proposed mechanism for the coupled physical adsorption/chemical photocatalysis degradation procedures of SO molecules is explained as follows:

$${\text{Cb}} - {\text{AgNPs}}\; + \;{\text{SO}}\;{\text{Dye}}\; \to \;{\text{Cb}} - {\text{AgNPs}}\;{-}{\text{SO}}\;{\text{Dye}}_{{{\text{adsorbed}}}} \left( {{\text{Sunlight}}} \right)$$

$${\text{Cb}} - {\text{AgNPs}}\;\left( {{\text{h}}^{ + } } \right){-}{\text{SO}}\;{\text{Dye}}_{{{\text{adsorbed}}}} + {\text{H}}_{{2}} {\text{O}} \to {\text{Cb}} - {\text{AgNPs}}\;({\text{OH}} \cdot ) - {\text{SO}}\;{\text{Dye}} + {\text{H}}^{ + }$$

$${\text{Cb}} - {\text{AgNPs}}\;\left( {{\text{h}}^{ + } } \right){-}{\text{SO}}\;{\text{Dye}}_{{{\text{adsorbed}}}} + {\text{OH}}^{ - } \to \;{\text{Cb}} - {\text{AgNPs}}\;\left( {{\text{OH}} \cdot } \right)\; - \;{\text{SO}}\;{\text{Dye}}$$

$${\text{Cb}} - {\text{AgNPs}}\;\left( {{\text{e}}^{ - } } \right)\;{-}{\text{SO}}\;{\text{Dye}}_{{{\text{adsorbed}}}} + \;{\text{O}}_{{2}} \to \;{\text{Cb}} - {\text{AgNPs}}\;\left( {{\text{O}}_{{2}} \cdot } \right) - {\text{SO}}\;{\text{Dye}}$$

$${\text{OH}} \cdot \;{\text{or}}\;{\text{O}}_{{2}}^{ - } \cdot + \;{\text{SO}}\;{\text{Dye}} - \,{\text{Cb}} - {\text{AgNPs }} \to \; \to \;{\text{Degraded}}\;{\text{Product}}\; + \;{\text{Free}}\;{\text{Cb}} - {\text{AgNPs}}\;{\text{for}}\,{\text{reuse}}$$

**Figure 7:**
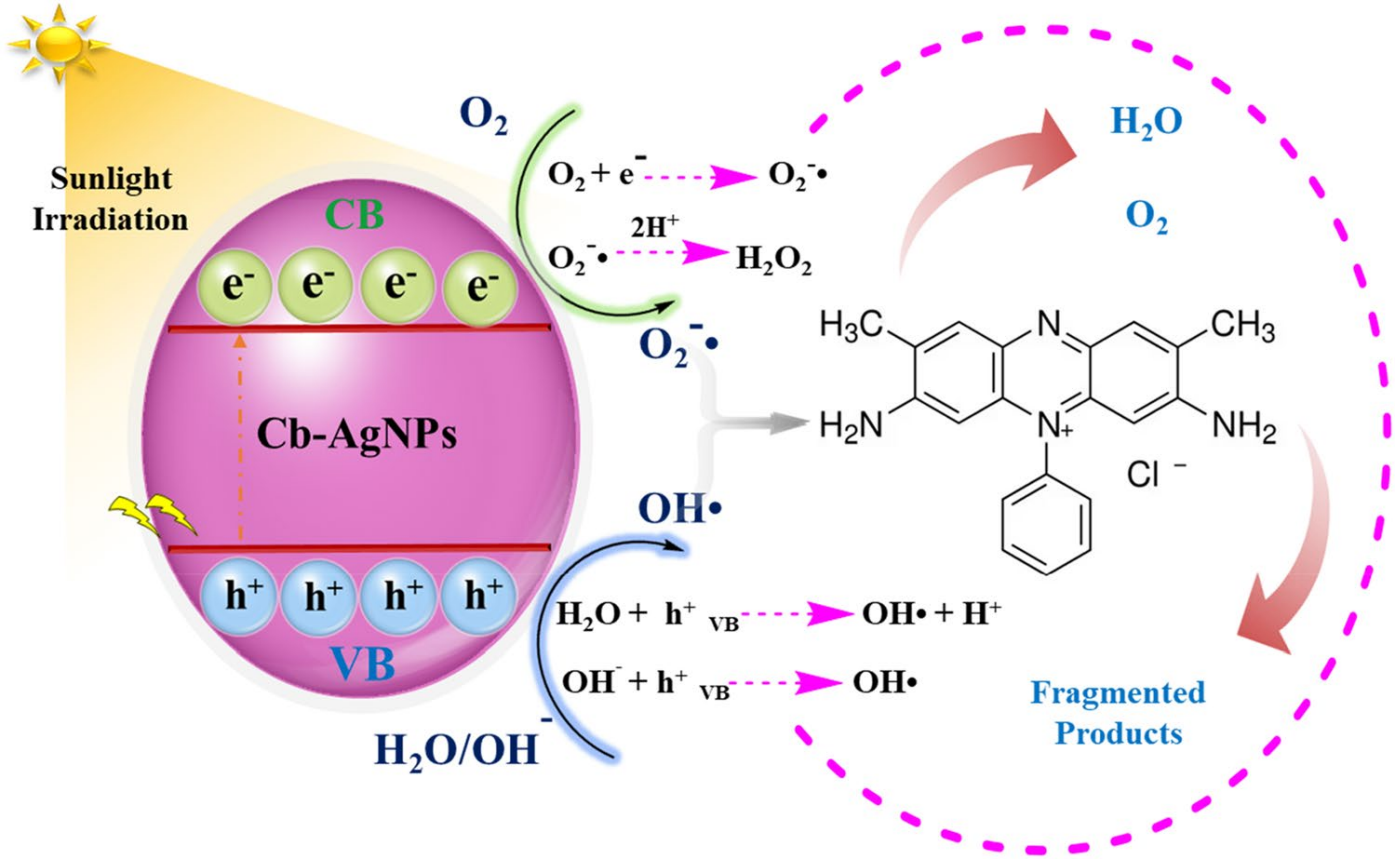
Proposed photocatalytic degradation mechanism of SO dye pollutant in the presence of Cb-AgNPs under sunlight irradiation.

The photodegradation process of SO using Cb-AgNPs, plotted by Ln A_0_/A_t_ versus irradiation time, indicated a linear correlation Fig. 6C. Thus, physical adsorption followed by photocatalysis of SO was fitted to pseudo-first-order kinetics model. Also, a linear correlation was observed between Ln A_0_/A_t_ and irradiation time Fig. 6D. The possible mechanism for the Cb-AgNPs photodegradation of dyes was proposed in Fig. 7.

The negative-charge of the Cb-AgNPs surface (− 26.6 mV) confirmed by zeta potential analysis indicates the potential of adsorption of cationic SO molecules on the surface of Cb-AgNPs.

In Table 2 the results obtained about the value rate constants (K_app_) of SO dye and regression coefficients (R^2^) are given. The comparison of apparent rate constants for physical adsorption followed by photocatalysis, and coupled physical adsorption/chemical photocatalysis undoubtedly indicated that the photocatalysis processes under synergistic conditions were more effective and proficient^70,71^.

**Table 2:** Removal percentage of SO by Cb-AgNPs under sunlight, rate constants, and linear coefficients from Ln A_0_/A_t_ versus Time plots.

Physical adsorption followed by photocatalysis Synergetic effect of physical adsorption/photocatalysis						
Degradation percentage in dark	Degradation percentage in sunlight	K_app_ (min^−1^)	R^2^	% Removal of dye in sunlight	K_app_ (min^−1^)	R^2^
42	68.33	0.0052	0.9819	98.47	0.0223	0.9782

### DPPH radical scavenging assay

**Figure 8:**
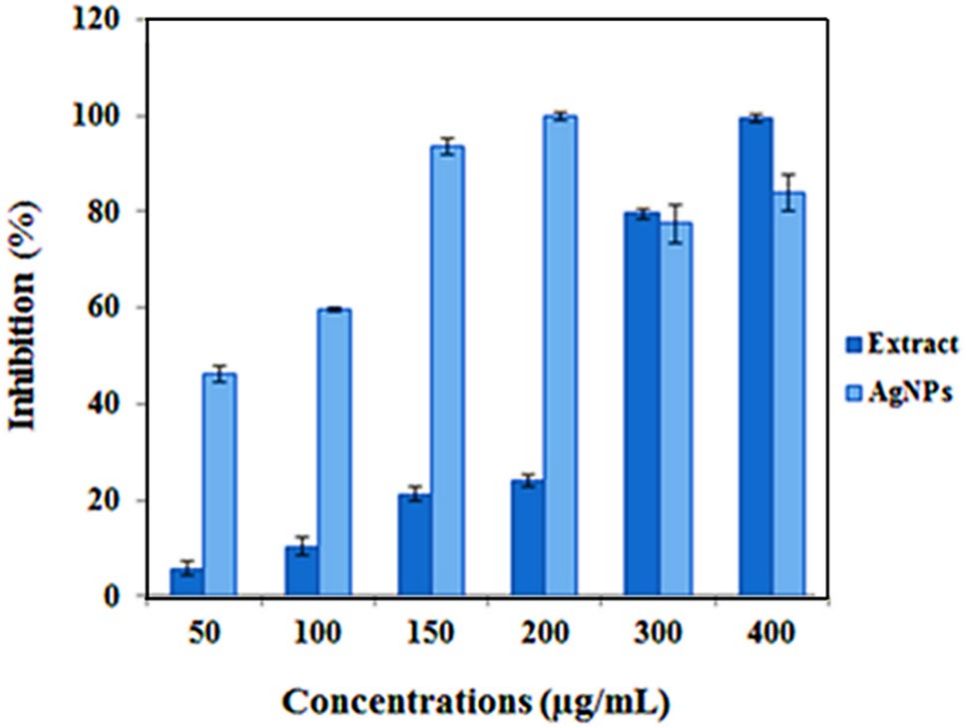
DPPH free radicals scavenging assays of phytogenic Cb-AgNPs.

The antioxidant activity of prepared phytogenic Cb-AgNPs and aqueous extract of *C. behen* at various concentrations (50, 100, 150, 200, 300, and 400 μg/mL) was assessed by, 2-diphenyl-1-picrylhydrazyl (DPPH) radical scavenging. As it has been shown in Fig. 8, the free radical scavenging activity of Cb-AgNPs in the concentration range of 50–200 μg/mL was higher than the aqueous extract and then its activity was decreased due to significant aggregation of nanoparticles at higher concentrations. On the other hand, DPPH radical scavenging ability of aqueous plant extract increased in a dose-dependent manner. The inhibition efficiencies of DPPH radicals by Cb-AgNPs from 50 to 200 μg/mL increased from 46.3 to 100%, while this parameter for aqueous plant extract at the same concentration range was from 5.8 to 99.5%. Cb-AgNPs have a large surface area which increased the adsorption of bioactive molecules, and for this reason their DPPH radicals scavenging ability are higher than the aqueous extract of *C. behen*.

### Antibacterial activity of Cb-AgNPs

**Figure 9:**
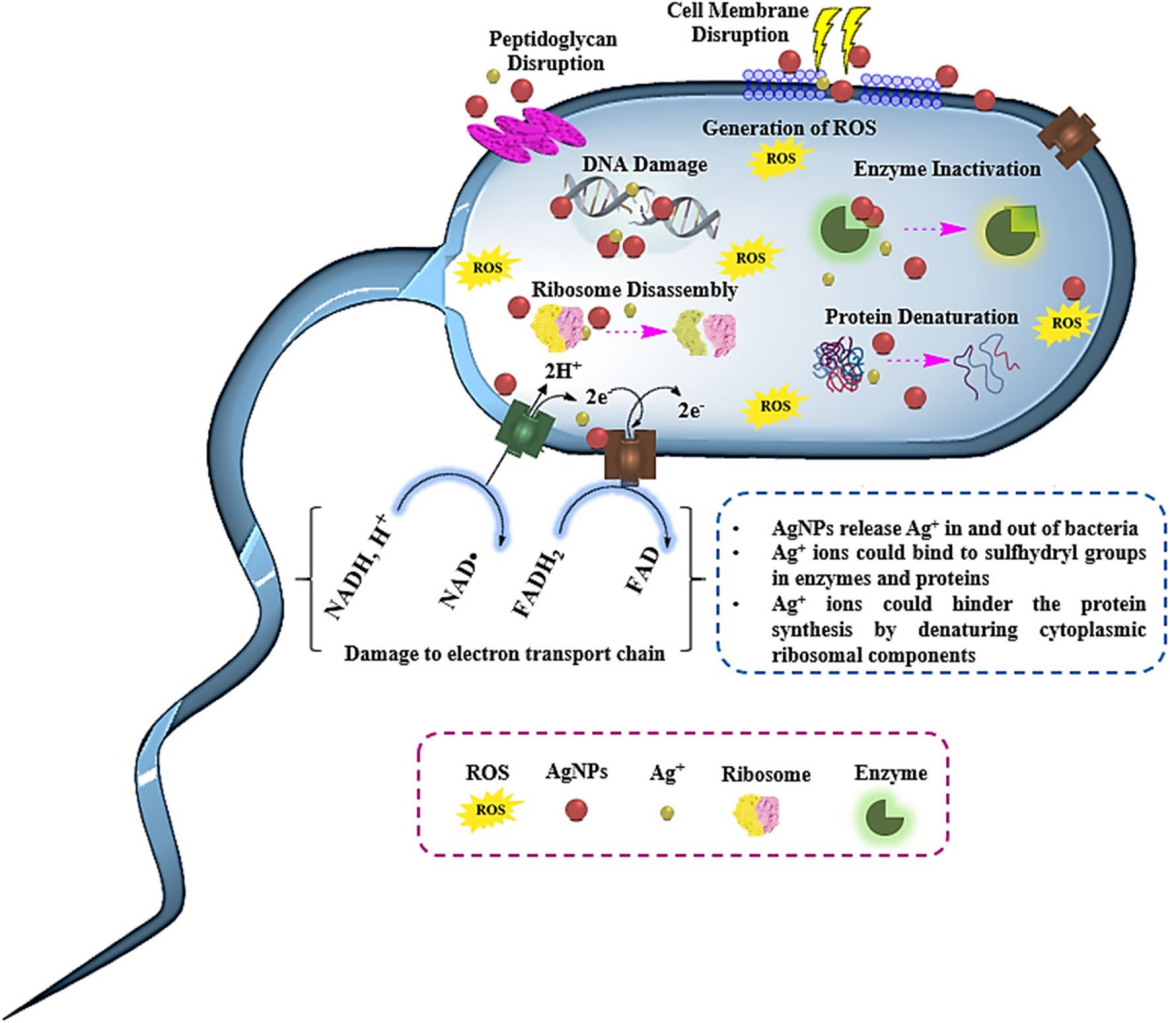
Schematic representation of AgNPs’ mechanism of action and their cellular targets.

Although AgNPs have shown effectiveness against various microorganisms, including bacteria, fungi, and viruses, the precise mechanism remains incompletely understood. However, two primary antimicrobial mechanisms have been identified: contact killing and ion-mediated killing^72^. In contact killing, AgNPs adhere to the cell wall surface and penetrate it, leading to membrane damage by binding to proteins in bacterial membranes. This damage results in the leakage of cellular contents and bacterial death (Fig. 9). Furthermore, AgNPs can access the bacterial cytoplasm after penetrating the membrane. The size-dependent efficacy of AgNPs in killing bacteria is notable, with smaller nanoparticles demonstrating higher antimicrobial activity due to their larger surface area, facilitating more frequent interaction with the cytoplasm compared to larger nanoparticles. Once within the cytoplasm, AgNPs interact with intracellular structures and biomolecules such as proteins, DNA, ribosomes, and enzymes, leading to damage and eventual cell death^73,74^.

Moreover, AgNPs can generate reactive oxygen species (ROS) as an additional antibacterial mechanism. Normally, ROS in cells are neutralized by antioxidant compounds like glutathione (GSH), but the presence of AgNPs may disrupt the expression of antioxidant enzymes. Elevated ROS levels can result in respiratory inhibition, reduced ATP production, apoptosis-like responses, lipid peroxidation, and DNA damage. Some studies suggest antibacterial mechanisms mediated by the release of Ag^+^ ions from AgNPs. The surface area of nanomaterials significantly influences the antimicrobial activity of AgNPs, with those possessing larger surface areas releasing higher concentrations of Ag^+^ ions into and out of cells. Silver ions bind to sulfhydryl groups in enzymes and proteins, including those responsible for transmembrane ATP generation, resulting in their deactivation. Additionally, Ag^+^ ions can inhibit respiratory chain enzymes, interact with DNA, impede cell division, and increase cellular oxidative stress^73,75^.

We determined the antibacterial actions of the Cb extract and Cb-AgNPs against four gram-positive bacteria strains including: *B. subtilis*, *S. epidermidis*, *S. aureus*, *M. luteus*, and also three gram-negative bacteria strains including: *E. coli*, *S. enterica* and *P. aeruginosa*. Finally, we measured the diameters of growth inhibition zones. As shown in Table 3, the Cb extract only affects the *S. aureus* bacteria strain at concentrations of 120 or higher. On the other hand, Cb-AgNPs affects all specified bacteria strains at different concentrations, except for *B. subtilis* (Table 4). Important key factors that determine the antimicrobial effects of nanoparticles are surface modification, concentration, purification methods and size of synthesized nanoparticles. Due to its larger surface area and better interact with bacteria pathogens, AgNPs have a specific antimicrobial property; AgNPs interact with membrane proteins and various cellular compounds such as phosphorus and sulfur in the cell. Since the electron transport chain components and the active transport system of ions and molecules are located in the bacterial plasma membrane, any change in the organization of the bacterial membrane leads to inhibition of bacterial growth. Also, AgNPs affect the respiratory enzymes, resulting in the production of reactive oxygen species which inhibit the bacterial growth. In addition, the size of nanoparticles has an important role in antimicrobial activity. Smaller nanoparticles have been shown to have more antibacterial effects^76,77^. Rajakumar et al. evaluated the effect of *Millettia pinnata* leaf extract and AgNPs on pathogenic bacteria. They showed that 10 μL of flower extract (10 mg/mL), AgNPs (5 and 10 mg/mL), had an antibacterial effect, but AgNPs have maximum growth inhibition effect^78^. In another study, the antibacterial effect of *Artemisia vulgaris* -AgNPs was evaluated against five human pathogenic bacteria (*E coli*, *S aurous*, *P aeruginosa*, *Klebsiella pneumonia* and *Haemophilus influenza*). Results showed that the *A. vulgaris* -AgNPs had inhibition effect against all bacteria with the highest value against S. aureus and the lowest value against *Asiatic cholera*^79^. Comparable findings were also reported by Thatoi et al.^80^. The present study revealed that the green prepared Cb-AgNPs show good antimicrobial activity against both gram positive and gram-negative organism.

**Table 3:** Zone of inhibition (mm) of (Cb-extract) for bacteria strains.

Bacteria strains		*S. aureus*	*S. epidermidis*	*B. subtilis*	*M. luteus*	*E. coli*	*Sal. enterica*	*P. aeruginosa*
Concentration	7.5	–	–	–	–	–	–	–
	15	–	–	–	–	–	–	–
	30	–	–	–	–	–	–	–
	60	–	–	–	–	–	–	–
	120	7 ± 0.82	–	–	–	–	–	–
	240	9 ± 0.12	–	–	–	–	–	–
Control (+)		24 ± 1	16.5 ± 0.55	17.5 ± 0.85	24.5 ± 0.75	26 ± 0.80	24.5 ± 1	25.5 ± 0.95

**Table 4:** Zone of inhibition (mm) of (Cb-AgNPs) for bacteria strains.

Bacteria strains		*S. aureus*	*S. epidermidis*	*B. subtilis*	*M. luteus*	*E. coli*	*Sal. enterica*	P. aeruginosa
Concentration	7.5	–	–	–	–	–	–	–
	15	–	–	–	8 ± 0.65	–	–	–
	30	8 ± 0.15	–	–	10 ± 0.33	–	7 ± 0.85	–
	60	9 ± 0.65	8 ± 0.22	–	12 ± 0.55	8 ± 0.82	8 ± 0.90	7 ± 0.70
	120	10 ± 0.75	9 ± 0.85	–	13 ± 0.84	9 ± 0.25	9 ± 0.88	8 ± 0.45
	240	11 ± 0.24	10 ± 0.15	–	15 ± 0.25	10 ± 0.12	10 ± 0.91	9 ± 0.26
Control (+)		24 ± 1	16.5 ± 0.55	17.5 ± 0.85	24.5 ± 0.75	26 ± 0.80	24.5 ± 1	25.5 ± 0.95

In this study we revealed that the Cb extract and Cb-AgNPs exhibited some degree of antibacterial activity at various concentrations, The Cb-AgNPs showed antibacterial activity at varied concentrations against *S. aureus*, *S. epidermidis*, *M. luteus*, *S. enterica*, *P. aeruginosa* and *E. coli*, but they did not show antibacterial activity against *B. subtilis* bacteria*,* at used concentrations. On the other hand, the Cb extract has antibacterial activity against *S. aureus*, at a certain concentration, but did not have antibacterial activity against other bacteria (Table 5).

**Table 5:** Antibacterial activity of Cb extract and Cb-AgNPs on gram-positive and gram-negative bacteria.

Agents	Methods	*S. aureus*	*S. epidermidis*	*B. subtilis*	*M. luteus*	*E. coli*	*Sal. enterica*	*P. aeruginosa*
Cb-extract	MIC (µg/mL)	120	–	–	–	–	–	–
	MBC (µg/mL)	240	–	–	–	–	–	–
Cb-AgNPs	MIC (µg/mL)	30	60	–	15	60	30	60
	MBC (µg/mL)	60	120	–	30	120	60	120

Compared to the present study, Slavin et al. indicated the greater antibacterial potential of lignin capped AgNPs with spherical shape and size about 20 nm against non-MDR species and MDR clinical isolates such as K. pneumonia, M. luteus, and P. aeruginosa with MIC values at a concentration between 5 and 25 µg/mL^81^. One of the main reasons for these researches can be attributed to the size of NPs, so smaller NPs tend to be more bactericidal compared to bigger NPs. However, the comparison of all MIC values reported in other studies is not possible owing to the variation of various parameters such as the precursor used s for the synthesis of NPs, the different origins of bacterial strains, the initial CFU used in the bacterial samples, the use of different reducing and capping agents in the synthesis process and the use of size of AgNPs with various size in the study^43,82^.

### Hemolytic activity

**Figure 10:**
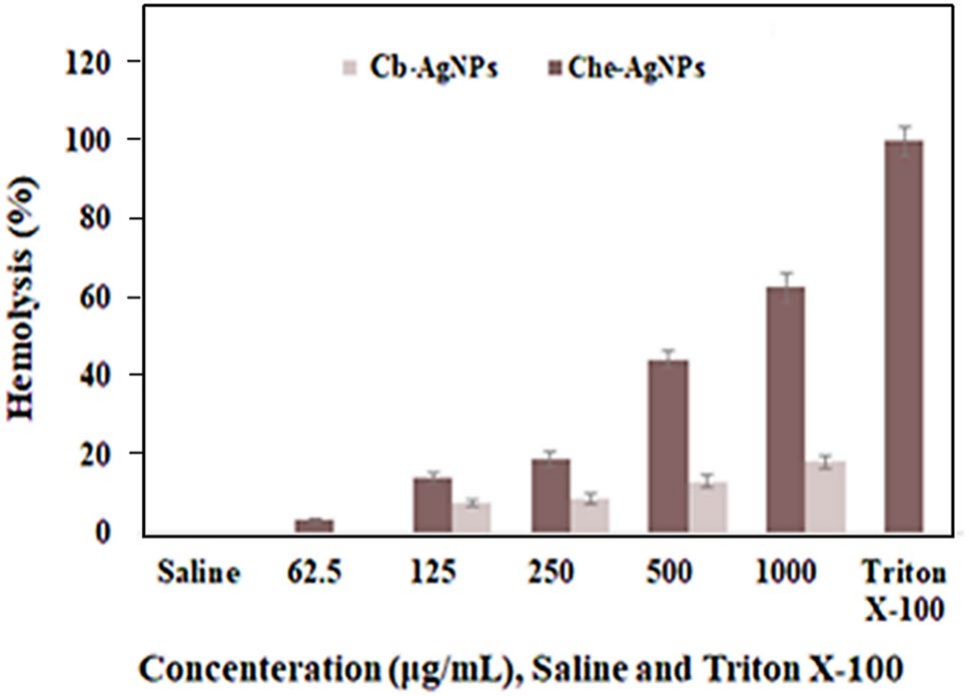
Diagram of hemolysis percentage, Normal saline as a negative control (1), Triton X-100 as a positive control (2), and Cb-AgNPs and Che-AgNPs at different concentrations of 25 (3), 50 (4), 125 (5), 250 (6), and 500 (7) μg/mL.

Some nanostructures may damage red blood cells and may rupture them and cause the release of hemoglobin^83,84^. Our findings showed that the extract at all concentrations and Cb-AgNPs solution at 25 μg/mL had no hemolytic activity, where at the same concentration, chemical synthesized-AgNPs (Che-AgNPs) significantly caused hemolysis (3.3%). Cb-AgNPs and Che-AgNPs both showed significant hemolytic activity at 25, 50, 125, 250 and 500 μg/mL, although in each concentration, the hemolytic activity of Cb-AgNPs was very less than the hemolytic activity of Che-AgNPs (*p* < 0.001) (Fig. 10). The hemolytic activity of Cb-AgNPs was 0, 7.6, 8.7, 13 and 18% compared to Che-AgNPs with 3.3, 14, 18.9, 44 and 62.5% hemolysis of RBCs at 25, 50, 125, 250 and 500 μg/mL, respectively. Compared to our result, Qasim Nasar et al.^85^ reported 70 and 99% hemolysis for AgNPs synthesized using leaf extracts of *Polyalthia longifolia* with a size range of 50–70 nm at concentrations of 50 and 100 μg/mL, respectively, which indicating better blood compatibility of Cb-AgNPs.

### Clotting time assay of Cb-AgNPs

**Figure 11:**
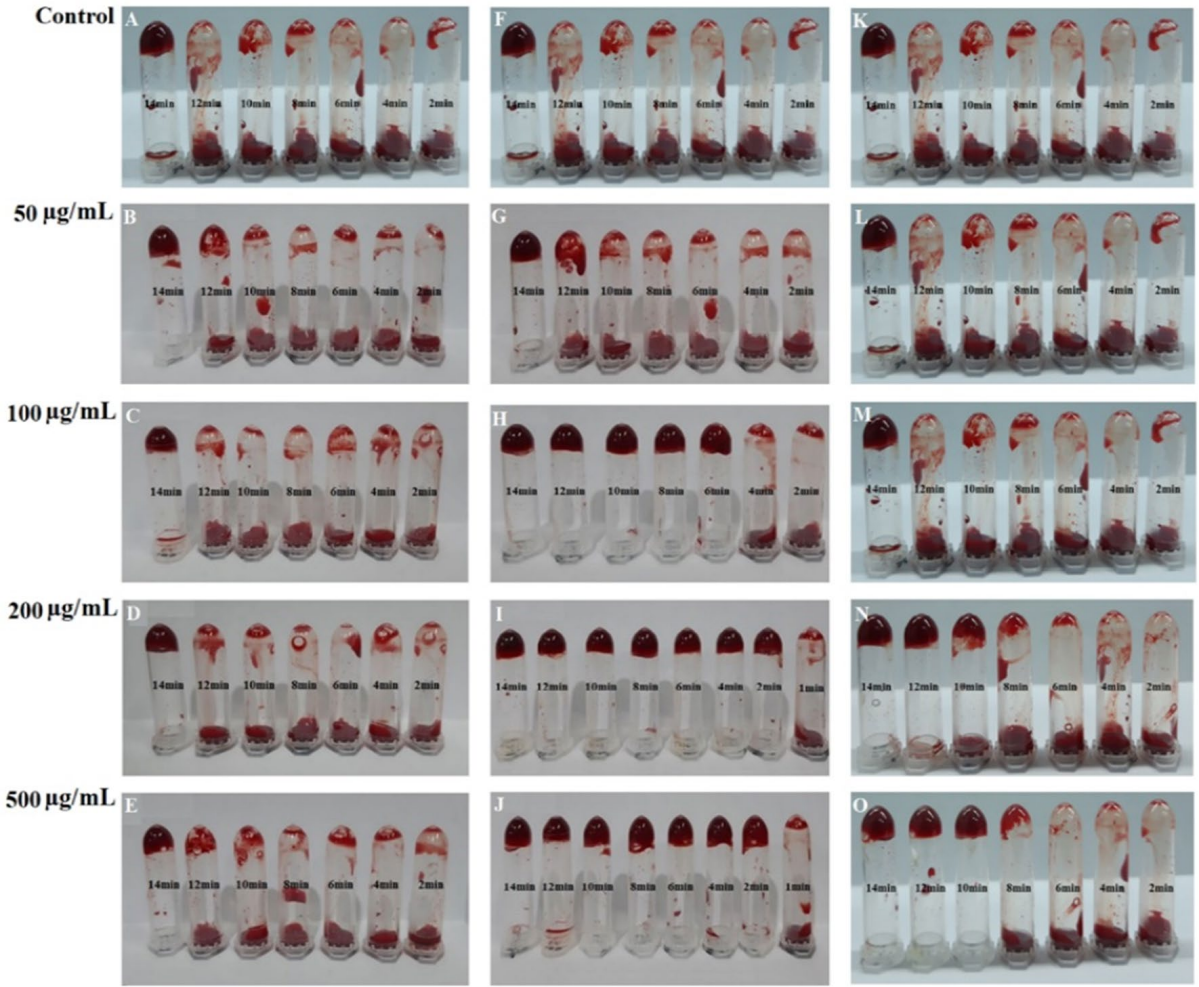
The in vitro clotting time assay of Cb extract (**A**–**E**), Cb- AgNPs (**F**–**J**) and Che- AgNPs (**K**–**O**) at concentrations: 50, 100, 200, 500 µ/mL and normal saline as control.

**Figure 12:**
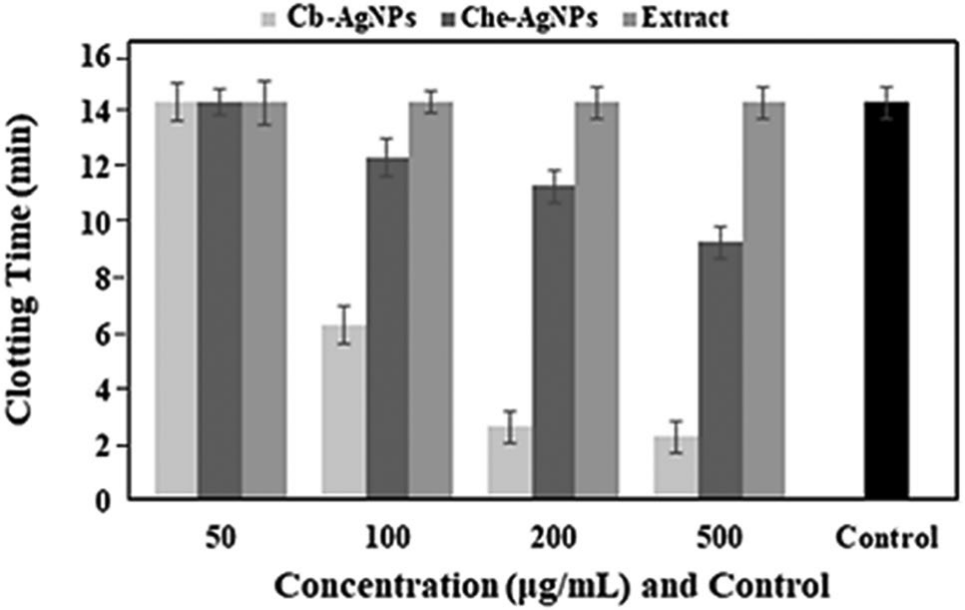
In vitro assessment of effects of concentrations 50, 100, 200, and 500 μg/mL from the Cb-extract, Cb-AgNPs, and Che-AgNPs solutions on the clotting time. The normal saline was used as control.

Wound and tissue healing is a four-stage, planned, overlapping, dynamic, and natural biological process in the body that includes homeostasis, inflammation, proliferation, and regeneration. Homeostasis is the process which stops bleeding through coagulation^86^. The results of an in vitro clotting time test showed that the concentrations of 50 μg/mL from Cb-AgNPs, 50 μg/mL from Che-AgNPs, and all the concentrations of the Cb-extract did not significantly alter the clotting time. As is shown in Figs. 11 and 12, the concentrations of 100, 200, and 500 μg/mL from Cb-AgNPs or Che-AgNPs decreased the clotting time significantly in comparison to controls and had coagulating effects (*p* < 0.05). Also, in these concentrations, the effects of Cb-AgNPs on the blood clotting time were more than the impact of Che-AgNPs (*p* < 0.001). Our results were confirmed by previous report by *Chahardoli* et al. (2020), which demonstrated the effective role of green synthesized AgNPs using *Achillea wilhelmsii* C. Koch in the reduction of blood clotting time (13 min compared to control)^87^. They explained that the decreased blood clotting time against AgNPs can be due to phytochemical of *A. wilhelmsii* extract as capping agents in the surface of biosynthesized AgNPs and also, negative charges of these NPs^87^. According to a previous report, the presence of high amount of flavonoid glycosides in the extract of some medicinal plants causes a potent hemostatic effect^88^. It has been reported that various species of the genus *Centaurea* have high amounts of flavonoids. The Cb extract also contains flavonoids such as flavone (crisimaritin and jeceosidin) and flavonoids of luteolin, salvegenin, and pectolinarigenin^89^. Therefore, the flavonoids in C. behen extract may contribute to the hemostatic potential of Cb-AgNPs and their ability to reduce clotting time. While Cb-AgNPs effectively decrease coagulation time, they also exhibit high hemolysis at elevated doses. However, lower concentrations could be suitable for wound dressings. Further research is needed to determine the absorption of these nanoparticles through the skin and their entry into the bloodstream.

## Conclusions

In this work, we applied the simple, rapid and inexpensive method for synthesis of AgNPs from an aqueous extract of *C. behen*. The physicochemical properties of obtained AgNPs were described by experimental analysis including UV–Vis, DLS, SEM, EDX, TEM, FTIR, and XRD. Most of the synthesized AgNPs have a spherical shape and size below 30 nm. The adsorption and catalytic degradation behaviors of SO dye onto Cb-AgNPs surface were investigated. The results reveal the synergistic effect of adsorption of SO dye on Cb-AgNPs surface helped the improving photocatalytic degradation process under sunlight irradiation These findings suggest that these NPs may applied for purification of Water polluted with industrial pollutants. The Cb-AgNPs inhibited the growth of *S. aureus*, *S. epidermidis*, *M. luteus*, *E. coli*, *Sal. enterica* and *P. aeruginosa* in a dose dependent manner. But at the desired concentrations, it had no effect on inhibiting *B. subtilis* growth. The elimination of microbial infection from a damaged site in the wounds and reducing large amounts of reactive oxygen species are important challenges in the wound healing process. Also, due to the fact that Cb-AgNPs prepared by green synthesis method have antioxidant and antibacterial properties and have a good ability to reduce coagulation time, they can be used to prepare wound dressings.

## Materials and methods

### Materials

Microorganisms used in this study represent pathogenic species commonly associated with infections, or important bacteria in research and medicine. The bacteria were maintained in the microbial Laboratory at, University of Kermanshah and consisted of *Staphylococcus epidermidis* (ATCC 12228), *Staphylococcus aureus* (ATCC 25923), *Bacillus subtilis* (ATCC 6633), *Micrococcus luteus* (*PTCC 9341*), *Salmonella enterica* (ATCC 9270), *Pseudomonas aeruginosa* (ATCC 27853), and *Escherichia coli* (ATCC 25922) that were supplied from Pasteur Institute of Iran. Silver nitrate salt (AgNO_3_), Safranin O, and methanol were bought from Merck, Germany. Mueller Hinton agar and broth medium, 1, 1-Diphenyl-2- picrylhydrazyl (DPPH) were provided from Sigma–Aldrich, USA.

### Processing of *Centaurea behen* leaves and determining its moisture content

Leaves of *C. behen* (Cb) was collected from near Divandarreh (Kurdistan Province, West Iran, 35°55′02.8″N 47°01′21.7″E). The plant was taxonomically identified and authenticated by Dr. Masoumi S.M. (Botany, Assistant Professor, Department of Biology, Faculty of Science, Razi University, Kermanshah, Iran 6714967346) and a voucher specimen (No: 2950 (HRU) /2023) was retained in the Herbarium of Razi University (HRU), Kermanshah, Iran for future reference and the plant has been maintained in an environmentally controlled greenhouse. Experimental research on the plant used for the study complies with relevant institutional, national, and international guidelines and legislation. The plant was washed frequently with Milli-Q water and dried thoroughly. To measure the MC, five of the fresh leaves of the plant were weighed and then heated in an oven at 110 °C for 3 h to constant weight. The samples were then cooled in a desiccator and re-weighed. The difference in weight of the initial sample with the dried sample is considered as the MC and is calculated according to the following formula:

$${\text{MC}}\;\left( \% \right) = \left( {{\text{Wet}}\;{\text{weight}} - {\text{Dry}}\;{\text{weight}}} \right)/{\text{Wet}}\;{\text{weight}} \times {1}00$$

### Preparation of extracts and synthesis of Cb-AgNPs

To synthesize of AgNPs, five gr of powdered leaves were blended in 100 mL of deionized water and sonicated for 30 min. The solution was passed through Whatman No. 1 filter paper and then centrifuged at 8800 rpm for 30 min, and the upper phase was utilized to synthesize AgNPs. Then, 10 mL of the extract was added to 100 mL of silver nitrate solution (1 mM). The reaction mixture was maintained to sunlight irradiation condition (34 °C, 34°24′20.8″N 46°43′54.5″E) until the conversion into reddish-brown color, which indicated the formation of AgNPs; then the reaction tube was centrifuged at 8800 rpm for 30 min. After three times washing with deionized water, the pellet was stored for further tests. Also, the AgNPs were prepared chemically using the method proposed by Fang, Zhang and Mu^90^ as a control. To synthesize AgNPs, a solution of Cb extract compound (2 mmol/L) was mixed with a solution of AgNO_3_ (1 mmol/L) in a 6:12 ratio. This mixture was then exposed to sunlight until a color change was observed. The transition from pale yellow to brown in the reaction solution indicated the formation of Cb -AgNPs.

### Characterization of Cb-AgNPs

The synthesized Cb-AgNPs were characterized by UV–vis spectrophotometer (Shimadzu UV 2450) in the wavelength ranging from 200 to 800 nm. The hydrodynamic size, PDI, and the zeta potential of AgNPs were evaluated by Zetasize analyzer (Zetasizer NS300, MALVERN, UK). The crystalline structure of the synthesized Cb-AgNPs was performed by X-ray diffraction (X'Pert Pro, Netherlands). Morphology of particle was analyzed by Transmission electron microscopy (TEM, Zeiss–LEO 906, Germany) at an accelerating voltage of 80 kV as well as Field Emission Scanning Electron Microscopy (TSCAN, Czech). FTIR Spectrophotometer (PerkinElmer Spectrum) over the frequency of 4000–400 cm^−1^ and at a resolution of 4 cm^−1^ was performed by the KBr pellet and transmission mode to determine functional groups involved in nanoparticles synthesis. Also, the identification of Cb-AgNPs was confirmed by using SEM, EDX and XRD.

### Photocatalytic studies

The photocatalytic activity of Cb-AgNPs, using SO as a cationic dye, was demonstrated under sunlight irradiation (34 °C, 34°24′20.8″N 46°43′54.5″E) by a somewhat changed method of Roy et al.^91^. Primarily, stock SO dye solution containing 100 mg/L of dye was prepared using double-distilled water. Effect of several process parameters like amount of photocatalyst, initial concentration of dye, solution pH, temperature and contact time on the extent of removal of SO dye were studied in detail. The influence of the pH of the initial solution was evaluated at pHs from 2 to 10 (adjusted using HCl (0.1 M) and NaOH (0.1 M)). Catalyst dosage was varied from 1 to 15 mg, keeping SO dye concentration at 10 mg/L. The influence of dye concentration using 5 mg of Cb-AgNPs photocatalyst was also evaluated by changing its concentration from 5 to 60 mg/L for SO dye.

Subsequently, 5 mg of Cb-AgNPs was added to 25 mL of dye solution (firstly, powder of Cb-AgNPs was dispersed in 5 mL DI water and sonicated for 20 min. Afterwards, it was added to 25 mL dye solution). At the same time, this colloidal suspension was stirred while it was exposed to natural sunlight irradiation. At certain intervals (every 15 min), 2 mL suspension was separated from the colloidal mixture and centrifuged at 5000 rpm for10 min to achieve clean supernatant of the checked SO dye. Eventually, the absorbance maxima of SO at different time intervals (0, 15, 30, 60, 90, and 120 min) was assessed using the UV–visible spectrophotometer at a wavelength range of 200–800 nm for estimation of dye adsorption/degradation.

The degradation rate (D) of the SO dye was calculated using Eq. (1):

1 $${\text{D}}\,\% = \left( {{\text{A}}_{0} - {\text{A}}_{{\text{t}}} } \right)/{\text{A}}_{0} \times {1}00$$

where A_0_ denotes the initial absorbance of SO solution(blank), and A_t_ its absorbance after t minutes of irradiation or reaction. According to the Beer_Lambert’s law A_0_ and A_t_ are proportional to C_0_ and C_t_, where C_0_ and C _t_ are the concentration of blank and sample at (t) time^56^.

### Radical scavenging activity

The antioxidant property of the crude extract and phytogenic Cb-AgNPs were estimated by using 1, DPPH assay. 2.5 mL of Cb extract and Cb-AgNPs at different concentrations (50, 100, 150, 200, 300, and 400 µg/mL in ethanol) was taken in separate test tubes; 1 mL of DPPH solution (0.3 mM in ethanol) was mixed with sample solutions and vortexed thoroughly. The reaction mixtures were incubated for 30 min in the dark condition at ambient temperature. Afterward, to calculate the percentage of inhibition of free radicals, the absorbance was assessed at 518 nm according to the Eq. (2):

2 $${\text{Inhibition}}\;{\text{of}}\;{\text{free}}\;{\text{radicals }}\left( \% \right):\left[ {\left( {{\text{Abs}}_{{{\text{sample}}}} {-}{\text{Abs}}_{{{\text{blank}}}} } \right)/{\text{Abs}}_{{{\text{control}}}} } \right] \times {1}00$$

Ethanol (1.0 mL) plus plant extract or phytogenic Cb-AgNPs solutions (2.5 mL) was used as a blank, and DPPH solution plus ethanol was used as a control^92^.

### Antibacterial assay

The total count of isolated strains of gram-positive (*B. subtilis*, *S. epidermidis*, *S. aureus*, and *M. luteus*) and gram-negative (*E. coli*, *S. enterica* and *P. aeruginosa*) bacteria were standardized to equivalent at 0.5 Mac Farland (1 × 10^8^ CFU/mL) by Mueller–Hinton broth. At first, the agar-disc diffusion method was applied to study antimicrobial activity. In this procedure, agar plates were inoculated with the inoculums of the above-mentioned bacteria. We prepared filter paper discs (6 mm in diameter) including a desired concentration of the drug compound (7.5, 15, 30, 60, 120 and 240 µg/mL), where standard antibiotic disc of gentamicin (10 μg) was used as the positive controls. Filter paper discs were placed on the agar surface and incubated at 37 ± 1 °C for 24 h. Then the diameters of inhibition zones were assessed.

In the next step antibacterial properties were estimated by broth dilution susceptibility assays. The final concentration of 1 × 108 CFU/mL bacterial strains was cultured into 96-well plates, and then 50 µL of Cb infusion or Cb-AgNPs at concentrations of 3.25, 7.5, 15, 30, 60, 120, and 240 µg/mL was added to wells and incubated at 37 ± 1 °C. After 24 h, the number of bacteria was recorded; the negative and positive controls were pure medium and the medium containing bacteria, respectively. One negative control well contained only pure medium and another negative control well contained pure medium and nanoparticles. On the other hands, positive control included only the pure medium and specific bacteria. The lowest concentration of Cb infusion or Cb-AgNPs, which is essential for inhibiting bacterial growth, was determined as the minimal inhibitory concentration (MIC). The minimum bactericidal concentration (MBC) is identified as the lowest concentration of Cb or Cb-AgNPs that killed 99.9% of the inoculums after 24 h incubation under standardized conditions in which there was after inoculation in plate agar no bacterial growth was seen^93^.

### Assay of hemolytic activity

The fresh human blood samples were obtained from the healthy volunteers (25–35 years old) and centrifuged at 800 g for 10 min; the red blood cells (RBCs) were separated and washed by using normal saline three times. Then, the suspension of RBCs in normal saline was prepared (10% v/v). Different concentrations of the Cb-AgNPs, chemical synthesized AgNPs (Che-AgNPs), and Cb extract (1000, 500, 250, 125, and 62.5 μg/mL) were made ready. 200 µL of the RBCs suspension was mixed with 200 µL of each concentration from sample solutions and was kept at 37 °C for 1 h. The final concentration of Cb-AgNPs, chemical synthesized AgNPs (Che- AgNPs), and Cb extract in the mixture were 500, 250, 125, 62.5, and 31.25 μg/ml. After centrifuging the samples at 13,400 rpm for 5 min, the supernatants were separated and the optical density (OD) of free hemoglobin released from RBCs into the supernatants were determined at 540 nm^94,95^. The hemolytic activity percentage of various concentrations of Cb-AgNPs was compared to the hemolytic activity of triton X100 as the positive control (100% hemolysis) and hemolytic activity of normal saline as the negative control (0% hemolysis) according to the following equation:

3 $${\text{Hemolytic}}\;{\text{activity}}\% = \left( {{\text{OD}}\;{\text{each}}\;{\text{concentration}}\;{\text{of}}\;{\text{NPs}} - {\text{OD}}\;{\text{negative}}\;{\text{control}}} \right)/\left( {{\text{OD}}\;{\text{positive}}\;{\text{control}}{-}{\text{OD}}\;{\text{negative}}\;{\text{control}}} \right) \times {1}00$$

The experiment was done in triplicates.

### Clotting time assay

Fresh human blood samples were collected from healthy voluntary individuals (25–35 years old) in 3.8% sodium citrate at a proportion of 1:9. Effects of different concentrations (50, 100, 200, 500 μg/mL) from the Cb-AgNPs, Che-AgNPs, and Cb extract solutions on the in vitro blood clotting time was assessed as described by previous methods^96^. 180 µL from citrated blood was added to 100 µL of each concentration of the solutions mentioned above or the normal saline as control and pipetted for 2 s. After that, to revoke anticoagulation effects of citrate sodium, the CaCl_2_ solution (0.1 M) was mixed with blood samples (at a ratio1:9) at particular times (0, 2, 4, 6, 8, 10, 12, 14), and clot forming was evaluated by inversion of the samples at min 14. The time that the mixture held its weight upon the tube inversion was noted as the clotting time. The experiment was done in triplicates.

### Statistics

One-way analysis of variance (ANOVA) was performed to compare the mean data using SPSS 16.0 software. *P* < 0.05 was considered significantly and the findings are given as mean ± standard error.

## Data Availability

The datasets used and/or analyzed during the current study available from the corresponding author on reasonable request.
